# Visual outcome is similar in optic neuritis patients treated with oral and i.v. high-dose methylprednisolone: a retrospective study on 56 patients

**DOI:** 10.1186/s12883-018-1165-6

**Published:** 2018-09-29

**Authors:** Magdalena Naumovska, Rafi Sheikh, Boel Bengtsson, Malin Malmsjö, Björn Hammar

**Affiliations:** 1 Department of Clinical Sciences Lund, Ophthalmology, Lund University, Skane University Hospital, Ögonklinik A Kioskgatan 1, SE-221 85 Lund, Sweden; 2 Department of Clinical Sciences Malmo, Ophthalmology, Lund University, Lund, Sweden

**Keywords:** Optic neuritis, Methylprednisolone, Corticosteroids, Multiple sclerosis

## Abstract

**Background:**

To investigate visual recovery after treatment of acute optic neuritis (ON) with either oral or intravenous high-dose methylprednisolone, in order to establish the best route of administration.

**Methods:**

Retrospective analysis of patients treated with oral or intravenous high-dose (≥500 mg per day) methylprednisolone for acute ON of unknown or demyelinating etiology. Twenty-eight patients were included in each treatment group. Visual acuity was measured with the Snellen letter chart, color vision with Boström-Kugelberg pseudo-isochromatic plates, and visual field with a Humphrey Field Analyzer.

**Results:**

The treatment results were similar in the two groups at follow-up, with no significant difference in visual acuity (*p* = 0.54), color vision (*p* = 0.18), visual field mean deviation (*p* = 0.39) or the number of highly significantly depressed test points (*p* = 0.46).

**Conclusions:**

The results show no clinical disadvantage of using oral high-dose corticosteroids compared to intravenous administration in the treatment of acute ON, which would facilitate the clinical management of these patients.

## Background

Optic neuritis (ON) is an inflammatory disease of the optic nerve. It typically manifests as subacute visual loss with pain that is often exacerbated by eye movement. ON is closely linked to multiple sclerosis (MS) and, in most cases, the pathogenesis is similar [1]. Approximately half the patients with ON will develop MS [1, 2].

Corticosteroids have been widely used for the treatment of MS relapse and optic neuritis, and the effect on short-term recovery of visual function has been well documented, while there are no long-term effects on visual outcome [3–8]. However, the best dosage, length of treatment and route of administration have not yet been established. Previous studies on the treatment of ON have compared the effect of a single route of administration of corticosteroids, either intravenous (i.v.) or oral, with a placebo [5–8], or unequally high doses have been compared [3, 9]. In the Optic Neuritis Treatment Trial (ONTT), a lower dose of oral prednisone was compared with a high i.v. dose of methylprednisolone (MP) [3]. The results suggested that high-dose i.v. MP increased the rate of recovery and visual function at six months, however, equally high doses of corticosteroids were not evaluated. The bioavailability of orally administered MP has been estimated to be 82% of that when MP is administered intravenously [10, 11]. Oral administration should therefore be of no disadvantage. Indeed, oral administration would facilitate the clinical management of these patients and is also safe, well-tolerated and less expensive than intravenously administered corticosteroids.

The aim of the present study was to compare the effects of oral and i.v. corticosteroids, both at high dose (≥500 mg MP per day), with regard to visual outcome in patients with acute ON. Twenty-eight patients were included in each treatment group. Visual acuity, color vision and visual fields were compared in the groups at follow-up.

## Methods

### Procedure

Patient records of subjects who were treated at the Department of Neurology and the Department of Ophthalmology at Skane University Hospital in Lund and Malmo, Sweden, between the years 2006 and 2017, were reviewed to identify patients with acute ON. According to local practice, all patients were first diagnosed with optic neuritis at the Department of Opthalmology and then treated at the Department of Neurology. Visual function was followed by an ophthalmologist. Patients were identified by searching the medical records for the diagnosis “optic neuritis”, “multiple sclerosis” and/or “retrobulbar neuritis”. By tradition, patients at the Lund clinic are more often treated with i.v. MP, whereas patients at the Malmo clinic more frequently receive oral MP regardless of the severity of symptoms. This is due to regional differences, in which the route of administration of corticosteroids is different but the treatment and follow-up is otherwise the same. In case of oral treatment, patients received methylprednisolone in tablet form for treatment at home, whereas those who were treated with intravenous corticosteroids were either hospitalized or had to visit the neurological department once daily to receive the treatment.

### Inclusion and exclusion criteria

Inclusion criteria were acute ON of unknown or demyelinating etiology in patients aged 18 years or older and treatment with high-dose MP (≥500 mg per day) orally or intravenously, without oral tapering. Exclusion criteria were previous ON in the same eye, repeated treatment with corticosteroids during the follow-up period, recent treatment (< 6 months) with corticosteroids for other complaints, neuromyelitis optica or systemic disease other than MS that might be the cause of the ON, initiated treatment with disease-modifying drugs in patients recently diagnosed with MS, and follow-up period less than 1 month or more than 6 months after the commencement of treatment. Patients were also excluded in cases when neuromyelitis optica was suspected, and aquaporin-4-antibodies were detected. Patients with MS who were already being treated with disease-modifying drugs prior to the advent of ON, and in whom this therapy was not changed, were not excluded. Patients with previous ON in the other eye were not excluded. In cases where data were available from several occasions during the follow-up period, the data closest to the six-month endpoint were chosen. Note that magnetic resonance imaging (MRI) pattern and cerebrospinal fluid oligoclonal band are not included in the current study as these tests were not reliably obtained in all subjects.

### Sample size

**Fig. 1:**
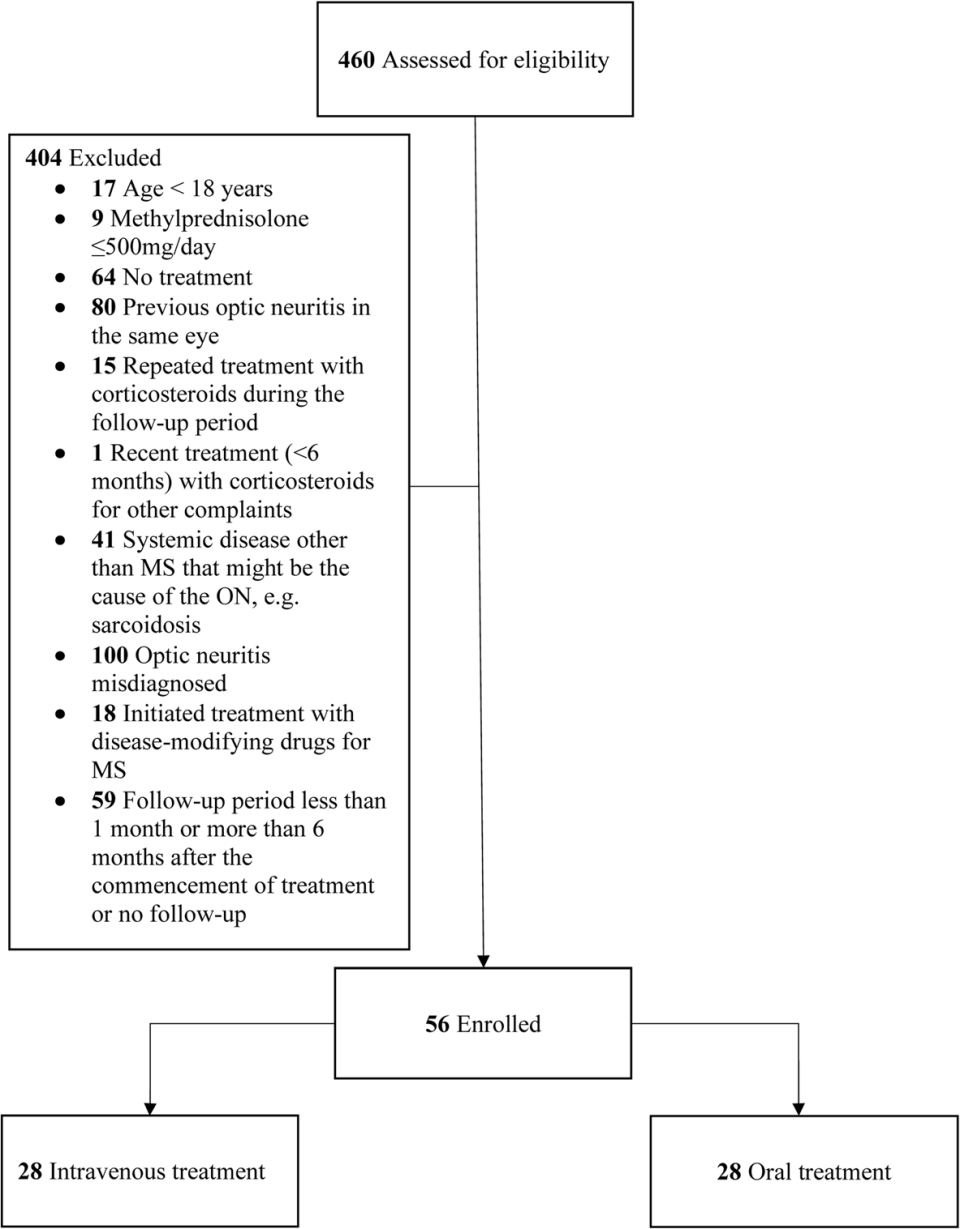
Flowchart of participant enrollment

Electronic patient records between the years 2006 and 2017 were reviewed in order to find patients with acute ON. Four hundred and sixty patients with suspected acute ON were assessed for eligibility and of these, 404 patients were excluded as a result of the inclusion and exclusion criteria. A total of 56 patients were included, with 28 subjects per treatment group. For participant enrollment, see Fig. 1.

### Patient characteristics

The two groups had similar durations of symptoms before the commencement of treatment and similar median follow-up times. They did not differ in total dose of MP or in treatment duration. Fewer patients had MS and were undergoing disease-modifying therapy for MS in the oral group than in the i.v. group before treatment for ON. There were no statistical differences in patient characteristics between groups. Data was not sufficient to explore side effects by treatment group. The detailed characteristics are given in Table 1.

**Table 1:** Patient characteristics

	All patients (*n* = 56)	Oral (*n* = 28)	i.v. (*n* = 28)
Gender (female/male)	42/14	20/8	22/6
Median age (range) (years)		33 (23 to 60)	35 (18 to 60)
Median duration of symptoms before treatment (range) (days)		7 (1 to 35)	7 (1 to 30)
Median total dose MP (range) (grams)		3.0 (1.5 to 5)	3.0 (2.8 to 6)
Median treatment duration (range) (days)		3 (3 to 5)	3 (3 to 6)
Median follow-up time (range) (weeks)		9 (4 to 24)	9 (4 to 24)
Multiple sclerosis before treatment (number of patients)	13	5	8
Undergoing disease-modifying therapy for MS (number of patients)	8	2	6
Previous ON in other eye (number of patients)	8	5	3

### Visual function measurements and calculations

The primary efficacy measures used were visual acuity, color vision and visual field outcome. Visual acuity was measured with the Snellen letter chart (Ortho-KM, Lund, Sweden), color vision with Boström-Kugelberg pseudo-isochromatic plates (BK) (KIFA, Stockholm, Sweden) [12], and visual field with a Humphrey Field Analyzer (HFA), SITA Standard program 30–2 or 24–2 (Carl Zeiss Meditec, Dublin, Calif, USA).

Decimal visual acuities were converted to logMAR units for statistical analysis. In cases where patients had poor visual acuity, hand movements were converted into a decimal visual acuity of 0.005, and finger counting was converted to 0.01 [13]. Color vision was described as the percentage of correct pseudo-isochromatic plates.

The results obtained with the HFA were expressed in two ways: the mean deviation (MD) in decibels, and the number of highly significantly depressed test points (DP) at the *p* < 0.005 level in the total deviation probability map. The total deviation probability map identifies and highlights test locations where the age-corrected threshold sensitivity is outside normal limits compared to healthy subjects. A highly significantly depressed test point indicates that 99.5% of normal subjects of the same age would be expected to have a sensitivity that is higher than the recorded value. This is considered to provide a more sensitive measure of visual field defects than the MD, as the MD is the weighted average measure of the deviations from the normal age-corrected threshold values of all test points in the visual field. To obtain the value of DP, HFA 24–2 was analyzed by counting and summing the number of highly significantly depressed test points. In subjects where HFA 30–2 was performed, only the test points corresponding to the HFA 24–2 program were included.

### Statistics

Results are presented as median values (range). Calculations and statistical analysis were performed using GraphPad Prism 7.0c and the Mann–Whitney test for comparisons (GraphPad Software Inc., San Diego, CA, USA). Significance was defined as *p* < 0.05.

## Results

**Fig. 2:**
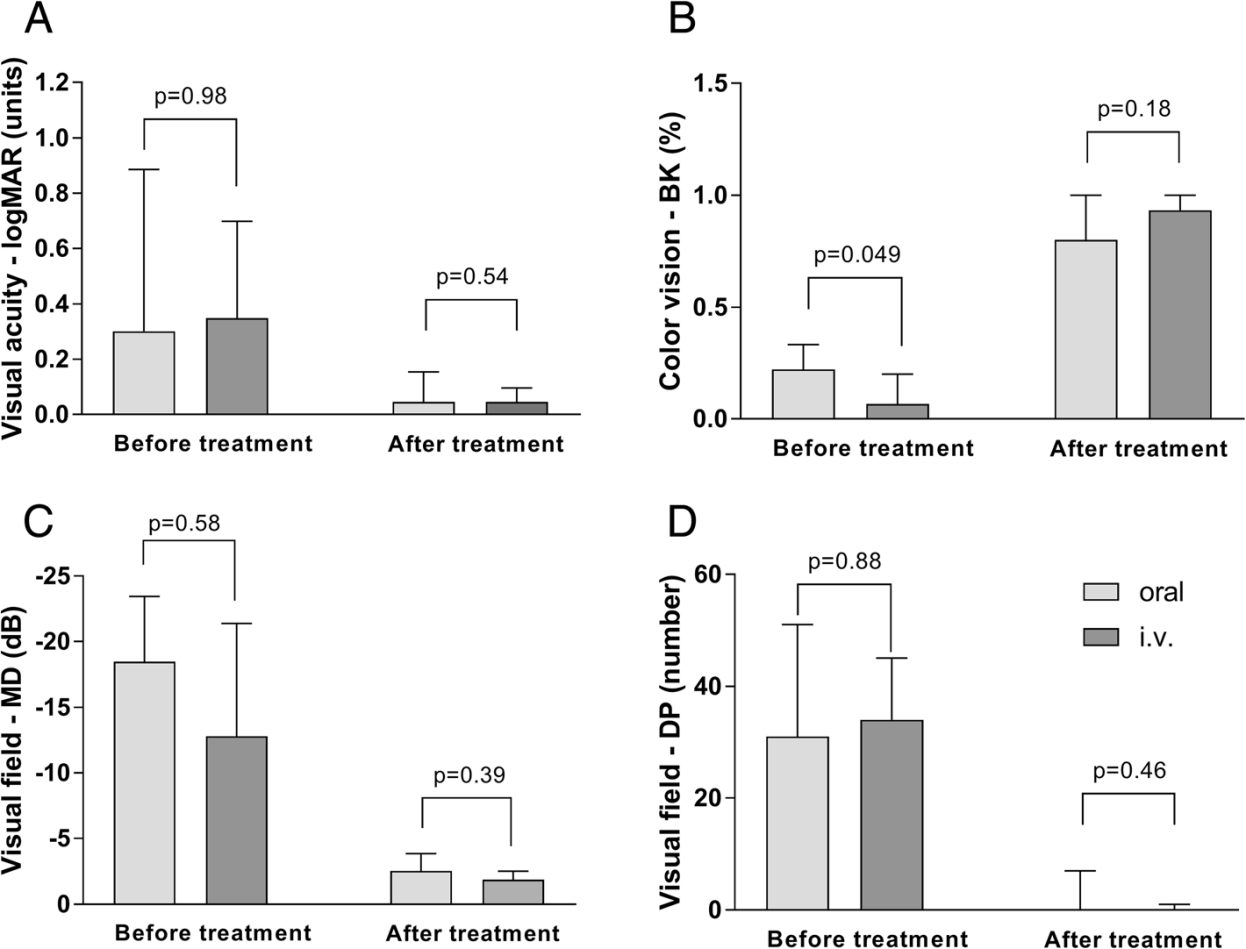
Results before and after treatment with oral or i.v. high-dose methylprednisolone. **a** Visual acuity expressed in logMAR units. **b** Color vision expressed as the percentage correct plates. **c** Visual field expressed as the mean deviation (MD) in decibels. **d** Visual field expressed as the number of highly significantly depressed test points (DP) in the total deviation probability map. Note that the visual outcome is similar in the two groups

**Fig. 3:**
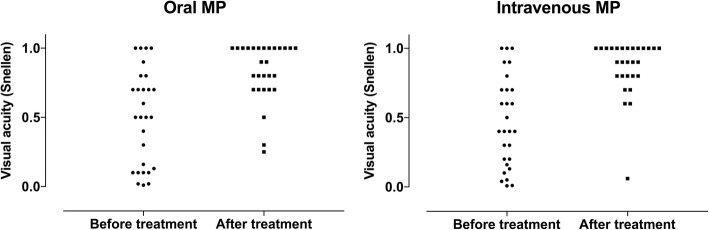
Visual acuity before and after treatment with oral or i.v. high-dose methylprednisolone, expressed in decimal visual acuity

The results of treatment at follow-up were similar in the two groups treated with oral and i.v. corticosteroids, showing no differences in visual acuity, color vision, visual field MD, or the number of highly significantly depressed test points. There was no difference in visual recovery between younger patients (age < 40 years) or older patients (age ≥ 40 years). See Table 2, Figs. 2 and 3 for detailed results.

**Table 2:** Results of treatment with oral versus i.v. high-dose methylprednisolone, expressed as median values (range)

	Before treatment			After treatment		
	Oral (*N* = 28)	i.v. (*N* = 28)	*p*-value	Oral (*N* = 28)	i.v. (*N* = 28)	*p*-value
Visual acuity, logMAR (units)	0.30 (0 to 2)	0.35 (0 to 2)	0.98	0.05 (0 to 0.60)	0.05 (0 to 1.22)	0.54
Color vision (percentage correct plates)	22.22 (0 to 100)	6.67 (0 to 100)	0.049	76.67 (0 to 100)	93.33 (13.33 to 100)	0.18
Visual field, MD (decibels)	−18.47 (−33.48 to −1.59)	−12.80 (−27.71 to −3.06)	0.58	−2.56 (−31.75 to −0.26)	−1.80 (− 11.4 to 0.48)	0.39
Number of highly significantly depressed test points in visual field	31 (0 to 52)	34 (2 to 52)	0.88	0 (0 to 52)	0 (0 to 45)	0.46

## Discussion

The results of this study show that there is no difference in visual outcome in patients with acute ON, treated with high-dose MP (≥500 mg per day) given orally or intravenously. Interestingly, in analogy with the present study on ON, others have studied the effect on MS relapses following high-dose oral corticosteroids, showing no inferiority of oral administration compared to i.v. administration, regarding MS disability outcome [14–16]. Furthermore, the results of our study are supported by a recently published prospective study on bioequivalent doses of corticosteroids in the treatment of acute ON, showing that oral administration of corticosteroids is not inferior to i.v. administration in terms of visual acuity and visual evoked potentials at follow-up [17]. It could be expected that the effects of oral and i.v. treatment would be similar as the bioavailability of oral MP has been reported to be as high as 82% of that given intravenously [11]. Interestingly, we found no difference in visual outcome, even though the oral group did not receive bioequivalent doses. Replacing high-dose i.v. administration of corticosteroids with oral administration would be of benefit to both the patient and the health care system. Indeed, the tolerability has been reported to be similar for both oral and i.v. administration of corticosteroids [15, 16].

In previous studies of oral versus i.v. administration of corticosteroids for the treatment of ON, equally high doses have not been tested, i.e. low-dose oral corticosteroids have been compared to high-dose i.v. corticosteroids. This is the case in the ONTT, in which the patients treated with oral corticosteroids received much lower doses of corticosteroids than those receiving i.v. treatment [3]. The rate of return of visual function was found to be higher following i.v. MP than with placebo, and the i.v. group exhibited slightly better visual field, contrast sensitivity, and color vision, but not better visual acuity, at 6 months. This was not found to be the case when oral administration of prednisone was compared to placebo. At 1 year, no difference was found between the groups, regardless of the route of administration [18].

In other studies, only a single route of administration of corticosteroids has been assessed, i.e. either i.v. or oral, and compared to the effect of a placebo. It has been reported that i.v. administration of corticosteroids increased the rate of recovery compared to placebo, but did not influence the final visual outcome [5–7], or the length of the lesion in the optic nerve [5]. Interestingly, in a study by Sellebjerg et al., the rate of recovery of visual function was improved in patients receiving high-dose MP orally compared to those given the placebo [8], showing the beneficial effects of high-dose oral steroids in the treatment of ON, supporting the findings in the present study.

As disease-modifying therapy may alter the course of recovery in acute demyelinating events [19, 20], such cases were excluded from the present study. However, patients undergoing therapy prior to the incident of ON were not excluded. The number of patients undergoing disease-modifying therapy was higher in the group receiving i.v. MP. The contributing effect of these agents on the course of recovery in acute demyelinating events has rarely been evaluated [21], and an additional effect can therefore not be ruled out. Prospective studies evaluating the effect of corticosteroids without disease-modifying therapy in the acute phase might be difficult in patients with demyelinating acute ON, as the criteria for the diagnosis of MS have changed during the past decade, and treatment with disease-modifying agents is now initiated early.

The test methods chosen to measure visual function were visual acuity, visual field and color vision, as these together provide a comprehensive picture of visual function. Regarding the visual field, a strength of our study is that we measured visual field defects by counting the number of highly significantly depressed test points in the total deviation probability map. This is likely to provide a more sensitive measure of visual field defects than the MD, since the latter is the average value of all deviations from the age-corrected normal threshold values of all test points in the visual field. We included the MD in our analysis to enable comparison with previous studies using MD as a measure of the visual field.

One limitation of the present study is the small number of subjects, which makes it difficult to draw definitive statistically supported conclusions. As this was a retrospective study, the data available in the patient records were also limited. For example, there was no information on visual evoked potentials (VEP) or Optical Coherence Tomography (OCT), as these are not standard tools for evaluating recovery after optic neuritis in a clinical setting at the departments included in the current study. For future prospective studies, this information would be of interest to analyze. Furthermore, the test used for color vision measurement is a non-specific test that is not optimized for the detection of acquired color vision deficiencies. A more suitable test should be used in future trials evaluating acquired color vision deficiency.

Regardless of whether ON is treated or not, the visual function starts to recover within 1 month [3, 22]. As ON improves spontaneously, treatment with corticosteroids has been questioned. A Cochrane review found that there was no evidence of any beneficial effect of oral or i.v. corticosteroids compared to placebo regarding visual acuity, visual field or contrast sensitivity outcomes [23]. However, even when visual acuity returns to normal, many patients have lasting symptoms of visual disability [24]. Optimal treatment should include the rapid relief of symptoms, as well as the prevention of tissue damage. Previous studies have shown that treatment with corticosteroids in ON has an effect on the rate of recovery and that the short-term risk of development of MS is reduced [25]. The effects of corticosteroid treatment have also been evaluated on brain MRI-derived quantities in MS, including gadolinium-enhancing lesions, showing a decrease in the number of lesions after treatment, also indicating the positive effect of corticosteroids [26, 27].

## Conclusions

The results of this study show no clinical disadvantage of using oral high-dose (≥500 mg MP per day) corticosteroids compared to intravenous administration in the treatment of acute ON. Oral corticosteroids are safe, well-tolerated, easy to administer and less expensive than i.v. corticosteroids. However, more prospective randomized trials must be carried out to evaluate the role of high-dose oral corticosteroids as a treatment option in ON before any clinical recommendations can be made.

## Abbreviations

BK: Boström-Kugelberg pseudo-isochromatic plates
DP: Number of highly significantly depressed test points
HFA: Humphrey field analyzer
i.v: Intravenous
MD: Mean deviation
MP: Methylprednisolone
MRI: Magnetic resonance imaging
MS: Multiple sclerosis
ON: Optic neuritis
ONTT: Optic Neuritis Treatment Trial
